# Cellular Tropism, Population Dynamics, Host Range and Taxonomic Status of an Aphid Secondary Symbiont, SMLS (*Sitobion miscanthi* L Type Symbiont)

**DOI:** 10.1371/journal.pone.0021944

**Published:** 2011-07-15

**Authors:** Tong Li, Jin-Hua Xiao, Zhao-Huan Xu, Robert W. Murphy, Da-Wei Huang

**Affiliations:** 1 Key Laboratory of Zoological Systematics and Evolution, Institute of Zoology, Chinese Academy of Sciences, Beijing, China; 2 Graduate School of the Chinese Academy of Sciences, Beijing, China; 3 College of Plant Protection, Shandong Agricultural University, Tai'an, Shandong, China; 4 State Key Laboratory of Genetic Resources and Evolution, Kunming Institute of Zoology, Chinese Academy of Sciences, Kunming, Yunnan, China; 5 Department of Natural History, Royal Ontario Museum, Toronto, Ontario, Canada; 6 College of Life Sciences, Hebei University, Baoding, Hebei, China; University of Wisconsin-Milwaukee, United States of America

## Abstract

SMLS (*Sitobion miscanthi* L type symbiont) is a newly reported aphid secondary symbiont. Phylogenetic evidence from molecular markers indicates that SMLS belongs to the Rickettsiaceae and has a sibling relationship with *Orientia tsutsugamushi*. A comparative analysis of *coxA* nucleotide sequences further supports recognition of SMLS as a new genus in the Rickettsiaceae. *In situ* hybridization reveals that SMLS is housed in both sheath cells and secondary bacteriocytes and it is also detected in aphid hemolymph. The population dynamics of SMLS differ from those of *Buchnera aphidicola* and titer levels of SMLS increase in older aphids. A survey of 13 other aphids reveals that SMLS only occurs in wheat-associated species.

## Introduction

Almost all aphids (Hemiptera: Aphididae) harbor the bacterial endosymbiont *Buchnera aphidicola*, which supplements essential amino acids lacking in the aphids' restricted diet of phloem sap [1]. The symbiont is harbored in specialized cells called bacteriocytes (or mycetocytes) that form an organ in the aphids' abdominal cavity called the bacteriome [2]. The bacterium is transmitted from mother to offspring with perfect fidelity, and the obligate relationship between *B. aphidicola* and aphids has been maintained for about 150–250 million years [3], [4].

In addition to *B. aphidicola*, aphids have about 12 vertically transmitted bacteria that are not essential for their survival. Most of these secondary or facultative symbionts are originally reported in *Acyrthosiphon pisum* [5], [6], [7], [8], [9], [10], [11], [12], [13], [14]. The three main secondary symbionts, *Serratia symbiotica* (R type), *Hamiltonella defensa* (T type) and *Regiella insecticola* (U type) endow aphids with diverse abilities such as resistance to high temperatures [15], parasitoid wasps [16], and fungal pathogens [17], and *R. insecticola* also can broaden the spectrum of host plants [18]. In contrast, symbiotic *Rickettsia* and *Spiroplasma* negatively affect the fitness of *A. pisum* [10], [19], [20]. Similar *in vivo* localizations of *S. symbiotica*, *H. defensa*, *R. insecticola* and *Rickettsia* occur in embryonic *A. pisum*; they are housed in sheath cells and secondary bacteriocytes around the primary bacteriocytes that contain *B. aphidicola*, as well as in aphid hemolymph [20], [21].

Recently, a new aphid secondary symbiont, SMLS (*Sitobion miscanthi* L type symbiont) was detected in *Sitobion miscanthi* and it probably represented a new genus in the family Rickettsiaceae [14]. Little taxonomic information was extracted from the *16S rRNA* sequence of SMLS. In present study, we investigated *in vivo* localization, population dynamics and host range, and clarified the taxonomic status of SMLS using *in situ* hybridization along with quantitative and diagnostic PCR techniques.

## Materials and Methods

### Ethics Statement

No experiment involving vertebrate samples was performed in this study. An ethics statement is not required for experiments that involve insects only. The collecting of wild aphids was permitted by wheat farmers.

### Materials

Aphids examined in this study were listed in Table 1. Previously, SMLS was detected in the population of *S. miscanthi* ZK collected from wild wheat in Zhoukou with a high frequency of infection (18/22, 81.8%). *Rickettsia* was detected in populations of *S. miscanthi* XN collected from wild wheat in Xining with a lower frequency of infection frequency (5/17, 29.4%) [14].

**Table 1:** Aphids examined in present study.

Aphid species (isofemale strain)	Collection locality	Total no. tested	SMLS^b^	*Rickettsia* c	Host plant
*Sitobion miscanthi* (ZK)	Henan, Zhoukou	NA^a^	+		Wheat
*Sitobion miscanthi*	Qinghai, Xining	17		5	Wheat
*Aphis spiraecola*	Ningxia, Liupanshan	20			Spiraea
*Aphis gossypii*	Ningxia, Liupanshan	5			Wormwood
*Aphis craccivora*	Liaoning, Xiuyan	8			Buckthorns
*Aphis eugeniae*	Guizhou, Mayanghe	2			Firethorn
*Aphis glycines*	Ningxia, Liupanshan	6			Soybean
*Toxoptera aurantii*	Guizhou, Mayanghe	5			Prickly ash
Toxoptera *odinae*	Hainan, Jianfengling	12			Chinese sumac
*Schizaphis graminum*	Shanxi, Taiyuan	10	1		Wheat
*Rhopalosiphum padi*	Hubei, Wuhan	5			Wheat
	Jiangsu, Nanjing	15	14		Wheat
	Beijing	5			Wheat
	Henan, Zhengzhou	18	8		Wheat
*Brachycaudus* sp.	Liaoning, Xiuyan	8			Buckthorns
*Chaitophorus populeti*	Beijing	10			Poplar tree
*Stomaphis* sp.	Beijing	6			Hickory nut
*Cinara* sp.	Ningxia, Jingyuan	6			Chinese pagoda tree

a Not applicable;

b, c detected SMLS and *Rickettsia* using *16S rRNA* specific PCR, the number represented positive samples.

SMLS = *Sitobion miscanthi* L type symbiont.

An *ex situ* SMLS-infected isofemale ZK-strain was built using one individual of *S. miscanthi* ZK. Aphids were reared on wheat seedlings in the laboratory at 20°C with a light∶dark regime of 16∶8 hr. Infections of the other three main secondary symbionts of aphids (*S. symbiotica*, *H. defensa* and *R. insecticola*) and the common symbiont of arthropods (*Wolbachia pipientis*) were tested in both the ZK-strain and *Rickettsia*-positive samples of the XN-population using *16S rRNA* diagnostic PCR [12], [22]. None of these symbionts was detected in ZK-strain and only one *Rickettsia*-positive sample in the XN-population was co-infected with *R. insecticola* (data not shown). Consequently, the gene amplifications of SMLS and *Rickettsia* were performed on the single-infection samples.

### DNA extraction, gene amplification, cloning, and sequencing

Total DNA was extracted from a single aphid using an EasyPure Genomic DNA Extraction Kit (TransGen, Beijing) following the manufacture's protocols. Aphid elongation factor-1α (*ef1α*) gene was used as a reference to evaluate DNA quality. The citrate synthase (*gltA*) and cytochrome C oxidase subunit I (*coxA*) genes of SMLS were amplified from the DNA of the aphid ZK-strain with forward primer gltAF3 (5′-ACATGCAGACCATGAGCAGA-3′) and reverse primer gltAR11 (5′-CATTTCATTCCATTGTGCCATC-3′), and forward primer coxAF1 (5′-GCTCCHGATRTKGCWTTTCC -3′) and reverse primer coxAR1 (5′-CATATTCCARCCDGCAAAAG -3′), respectively. Both gene fragments were amplified from the DNA of *Rickettsia*-positive samples of the XN-population, with forward primer gltAF11 (5′-GGGTTTTATGTCTACTGCTTCTTG-3′) [23] and reverse primer gltAR11, and forward primer coxAF4 (5′-TTTACTGCCGGYWCAATGAT-3′) and reverse primer coxAR1, respectively. Cycling conditions were 94°C for 4 min, followed by 38 cycles at 94°C for 30 s, 53°C for 45 s, 72°C for 1 min, and a final elongation for 10 min. PCR products were purified using an EasyPure PCR Purification Kit (TransGen), and cloned with the pEASY-T1 vector (TransGen). Three positive clones of each amplicon were sequenced.

### Molecular phylogenetic analysis

To reveal the phylogenetic position of SMLS within the Rickettsiales, nucleotide sequences of *16S rRNA*, *gltA* and *coxA* representing the two main families were retrieved from GenBank for the following taxa: family Anaplasmataceae (*Anaplasma marginale* [NC_012026], *Ehrlichia ruminantium* [NC_006831], *Neorickettsia risticii* [NC_013009], *Wolbachia pipientis* [NC_010981]); family Rickettsiaceae (*Rickettsia bellii* [NC_007940] , *Rickettsia prowazekii* [NC_000963], *Rickettsia rickettsii* [NC_010263], *Rickettsia typhi* [NC_006142], *Orientia tsutsugamushi* [NC_009488]). Based on the phylogenetic tree for the alphaproteobacteria [24], we chose two species from the Rhodospirillales, *Acidiphilium cryptum* [NC_009484] and *Gluconacetobacter diazotrophicus* [NC_011365], as the outgroup.

Sequences were initially aligned using Clustal W as implemented in MEGA 4.0 [25] with the default parameters and then adjusted manually. Bayesian inference (BI) trees were constructed in MrBayes 3.1.2 [26], [27]. The best-fit nucleotide substitution models were selected using jModelTest 0.1.1 [28], [29] based on Akaike Information Criterion [30]. Two independent runs including four chains were performed with initial 1,000,000 generations, and stopped when the average deviation of split frequencies fell well below 0.01. Trees were sampled every 100 generations and the initial 25% of the total trees were discarded as burn-in. Compatible groups were shown in the majority rule consensus tree. Analyses involved independent gene and the concatenated data. In the latter case, the concatenated data were partitioned as independent gene. The parameters were defined as unlinked and the prior rate was set as variable. Branch support for each node in BI trees was assessed by the frequency of nodal resolution, i.e., a Bayesian posterior probability (BPP).

### Fluorescence *in situ* hybridization

This process was generally performed as described by Koga et al. [31]. Aphid embryos were dissected from adults of the ZK-strain in cold 70% ethanol using the hooked tip of a #0 insect pin (0.3 mm diameter, 40 mm length) under a stereoscopic microscope, and then fixed in Carnoy's solution (chloroform-ethanol-acetic acid [6∶3∶1]) for 10 hr. The fixed embryos were decolorized overnight in alcoholic 6% H_2_O_2_ solution, then pre-hybridized in hybridization buffer (20 mM Tris-HCl [pH 8.0], 0.9 M NaCl, 0.01% sodium dodecyl sulfate, 30% formamide) for 3 times at 6 hr each. Embryos were then incubated overnight in hybridization buffer containing 100 pmol/ml of each fluorescent probe and 0.5 µg/ml 4′,6′-diamino-2-phenylindole (DAPI). Finally, the embryos were washed in a buffer (0.3 M NaCl, 0.03 M sodium citrate, 0.01% sodium dodecyl sulfate) and observed under a laser confocal microscope (LSM 510 META, Carl Zeiss). We designed two fluorescent probes that targeted *B. aphidicola* and SMLS 16S rRNA molecules in cells from known probes [20]: SMB-Cy5 (5′-Cy5-CCTCTTTTGGGTAGATCC-3′) for *B. aphidicola*, and SMLS-Cy3 (5′-Cy3-TCCACGTCACCGTATTGC-3′) for SMLS. Nuclei of aphid cells were counterstained with DAPI. No-probe and RNase digestion control experiments were employed to confirm the specificity of the detection. All manipulations were performed at room temperature.

### SMLS detection in aphid hemolymph

Aphid hemolymph was collected from about 10 adult aphids of the ZK-strain following the methods described by Fukatsu et al. [7]. Bacterial DNA was extracted using the same DNA extraction kit. SMLS was detected with *16S rRNA* diagnostic PCR, using the following primers: 16SA1 (5′-AGAGTTTGATCMTGGCTCAG-3′) [32] and Ric16SR (5′-TCCACGTCACCGTCTTGC-3′) [20]. Aphid DNA of the ZK-strain served as the positive control, and sterile water used as the template in the negative control.

### Quantitative PCR

DNA was extracted from a series of aphids of the ZK-strain according to days after birth. Titers of SMLS and *B. aphidicola* were quantified in terms of the *gltA* gene and molecular chaperone dnaK (*dnaK*) gene copies, respectively. Quantitative PCR was performed in a Mx3000 (Stratagene, USA) using the SYBR Green I method. Primers were designed by the online program Primer3 (v. 0.4.0) [33]. Forward primer SMLS-gltAqF (5′-AGAATCAAGAATATGTGACAATC-3′) and reverse primer SMLS-gltAqR (5′- CCATCCAACCACTAACAC-3′) amplified a 200 base-pair (bp) fragment of *gltA* with about 98.9% amplification efficiency (estimated in standards). Forward primer Buch-dnaKqF (5′-AAGCAGTTATTACAGTTC-3′) and reverse primer Buch-dnaKqR (5′-GCTATTGTTCTATTACCTT-3′) amplified a 163 bp fragment of *dnaK* with about 94.0% amplification efficiency. Aphid cell concentration was quantified in terms of *ef1α* gene copies, and forward primer ef1αqF (5′-GCACCTGGACATAGAGATT-3′) and reverse primer ef1αqR (5′-GACAATAAGCACAGCACAA-3′) amplified a 75 bp fragment of *ef1α* with about 98.6% amplification efficiency. Three pairs of primers had high amplification specificity as verified by unique peaks observed in respective melting curves (data not shown). Quantitative PCR reactions were carried out in a 25 µl volume containing 12.5 µl 2×TransStart Green qPCR SuperMix UDG (TransGen), 0.5 µl 50×Passive Reference Dye, 10 µl sterile water, 0.5 µl of each primer (10 µmol) and 1 µl DNA. Cycling conditions were 50°C for 2 min (UDG enzyme digestion), 94°C for 10 min, followed by 35 cycles at 94°C for 30 s, (55°C for *gltA* and *ef1α*, 53°C for *dnaK*) for 30 s, 72°C for 30 s. Finally, a melting curve was constructed. Standard curves were constructed with serial dilution plasmids, which contained 10^10^, 10^9^, 10^8^, 10^7^, 10^6^ and 10^5^ copies/µl of *gltA* and *dnaK*, 10^8^, 10^7^, 10^6^, 10^5^ and 10^4^ copies/µl of *ef1α*. Sterile water was used as the template in the NTC (no template control).

### Diagnostic PCR

*Rickettsia* and SMLS were detected within diverse species of aphids (Table 1) using *16S rRNA* diagnostic PCR. Cycling conditions were the same as those used in the amplification of *gltA* and *coxA*. DNA of the aphid ZK-strain was used as the template in the positive control, and sterile water was used as the template in the negative control.

### Nucleotide sequence accession numbers

The *gltA* and *coxA* sequences of SMLS, and *Rickettsia* from *S. miscanthi*, were deposited in GenBank under accession numbers HQ645970–HQ645973. The *16S rRNA* sequences of SMLS isolated from *Schizaphis graminum* and *Rhopalosiphum padi* were deposited in GenBank under accession numbers JF933898–JF933900.

## Results

### Amplification and identification of the *gltA* and *coxA* sequences

Putative *gltA* and *coxA* sequences of SMLS and *Rickettsia* from *S. miscanthi* were amplified, cloned and sequenced. A 475 bp fragment (excluding primer sequences) was obtained with primers gltAF3 and gltAR11 for the ZK-strain and it was most similar (70%) to that of *R. typhi* when searched by Blastn in NCBI (http://www.ncbi.nih.gov/BLAST/). A 1049 bp fragment was obtained with primers coxAF1 and coxAR1 for the ZK-strain. It was 80% similar to the sequence of *O. tsutsugamushi*. The primers gltAF11 and gltAR11 amplified a 1023 bp fragment from the DNA of *Rickettsia*-positive *S. miscanthi*. The fragment was 97% similar with that of *R. bellii*. Finally, a 404 bp fragment was obtained from *Rickettsia*-positive *S. miscanthi* with primers coxAF4 and coxAR1 and it was most similar (96%) to *R. bellii*. All sequences were converted into amino acids to confirm translation.

### Phylogeny

**Figure 1:**
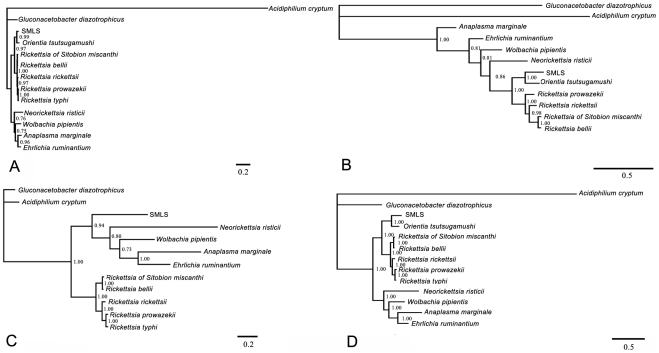
Bayesian inference trees were constructed with individual gene and the concatenated data of all loci. A, *16S rRNA* tree; B, *coxA* gene tree; C, *gltA* gene tree; D, concatenated data tree. Numbers near nodes indicate Bayesian posterior probabilities >0.5. The bar indicates the estimated number of substitutions per site.

Using jModelTest, the GTR substitution model with rate variation among sites (+G) was selected for *16S rRNA* and *coxA*, and TIM3+G was selected for *gltA*. The parameters of these models were estimated in MrBayes. In addition to BI trees, we also constructed neighbor joining (NJ) trees with Kimura 2-parameter substitution model in MEGA 4.0, and searched maximum parsimony (MP) trees with heuristic method, TBR algorithm in PAUP 4.0b10* [34]. The three methods obtained almost identical topologies for *16S rRNA* and *coxA*. Moreover, BI obtained better resolution than NJ and MP when using *gltA* and the concatenated data. Herein, we only provided the BI trees (Figure 1). Monophyly of the Rickettsiales was supported in all analyses, although not highly supported using *16S rRNA* alone (BPP<0.5). The monophyly of Rickettsiaceae was supported in all analyses, while the monophyly of Anaplasmataceae was not supported in the *coxA* gene tree. SMLS usually clustered with *O. tsutsugamushi* in the Rickettsiaceae. However, SMLS also clustered with the Anaplasmataceae in the *gltA* tree, in which *O. tsutsugamushi* was not included. The close affinity between *Rickettsia* from *S. miscanthi* and *R. bellii* was highly supported in all analyses.

### *In situ* hybridization of SMLS

**Figure 2:**
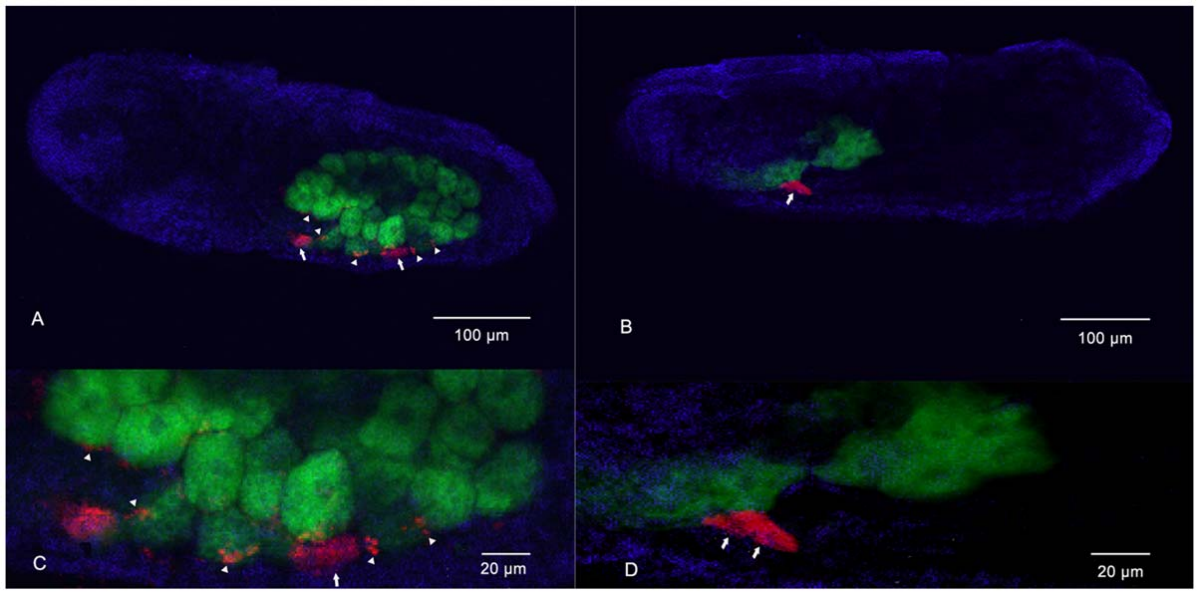
Whole-mount *in situ* hybridization of aphid embryos. *Buchnera aphidicola* (green), SMLS (red) and nuclei of aphid cell (blue). A, B, sheath cells and secondary bacteriocytes harbouring SMLS and primary bacteriocytes harbouring *B. aphidicola*. C, D, magnified images of A and B. Arrows, secondary mycetocytes; arrowheads, sheath cells.

Two patterns of infection were observed in cells (Figure 2): low density SMLS harbored in the sheath cells (Figure 2A, C; arrowhead) and high density SMLS in secondary bacteriocytes, cells larger than sheath cells (Figure 2C, D; arrow). Some secondary bacteriocytes intercalated between primary bacteriocytes (Figure 2C, arrow). Control experiments (no-probe and RNase digestion) confirmed the specificity of the observed signals (data not shown).

### SMLS detection in aphid hemolymph

SMLS was detected in hemolymph. No product was amplified in negative controls, removing the possibility of contamination during amplification.

### Population dynamics of SMLS and *B. aphidicola*

**Figure 3:**
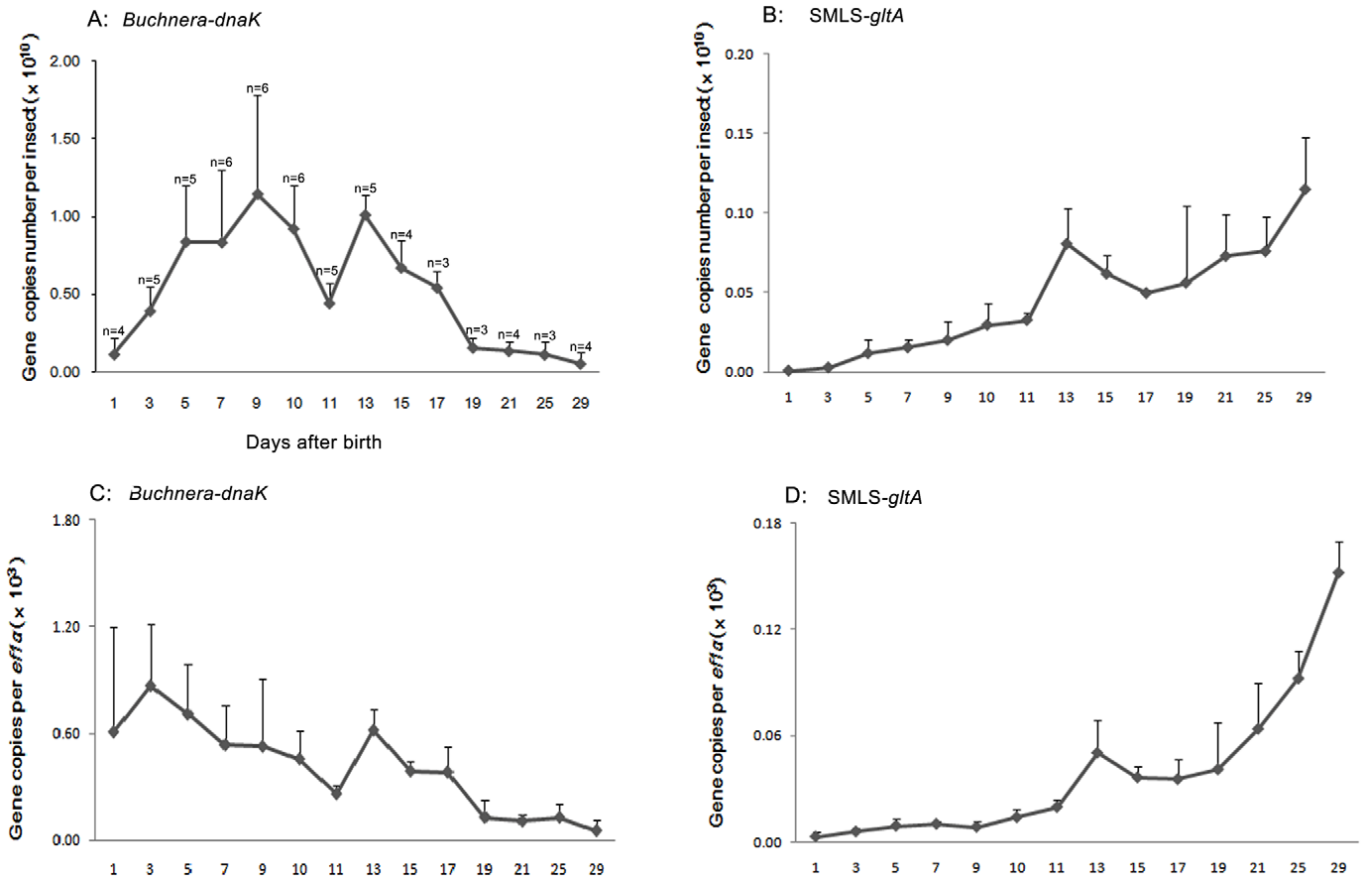
Population dynamics of *Buchnera aphidicola*, SMLS along with the development of *Sitobion miscanthi*. A, population dynamics of *Buchnera aphidicola* in terms of *dnaK* copies; B, population dynamics of SMLS in terms of *gltA* copies; C, density dynamics of *Buchnera aphidicola* in terms of *dnaK* copies per *ef1α* copy; D, density dynamics of SMLS in terms of *gltA* copies per *ef1α* copy. Means and positive standard deviations shown; numbers near bars show the replicates.

The quantitative PCR results (Figure 3) revealed that the population of *B. aphidicola* (Figure 3A) increased during nymphal growth, peaked at the 9 day-stage when aphids matured, declined in the active reproduction day-stages (from 9 to11 day-stages), resurged at the 13 day-stage, and declined again in the remaining stages (from 13 to 29 day-stages). When normalized by titers of the host gene (*ef1α*), the density of *B. aphidicola* (Figure 3C) exhibited similar dynamics but declined from 5 to 9 day-stages. The population of SMLS (Figure 3B) increased from 1 to 13 day-stages, declined from 13 to17 day-stages, then increased again to attain its highest density in the 29 day-stage. When normalized by the titers of host gene (Figure 3D), the density of SMLS exhibited the same dynamics.

### Diagnostic PCR of *16S rRNA*

To estimate the incidence of *Rickettsia* and SMLS infections across species of aphids, 141 samples of 13 species of aphids were subjected to diagnostic PCR for *16S rRNA*. Taken together with the sequencing results, none of these aphids appeared to be infected with *Rickettsia* but SMLS was detected in *S. graminum* and *R. padi* with infection rates of 10% and 51.2%, respectively.

## Discussion

In the *gltA* tree, *O. tsutsugamushi* was not included because the species lost its functional *gltA* gene [35]. SMLS clustered with the Anaplasmataceae, perhaps due to long branch-attraction or repulsion. Considering the robust supports in phylogenetic analyses of *16S rRNA*, *coxA* and the concatenated data, SMLS most likely belonged to the Rickettsiaceae and had a sibling relationship with *O. tsutsugamushi*. The high level of sequence divergence (6%) between *16S rRNA* from *O. tsutsugamushi* and SMLS previously indicated that SMLS might best be classified as a new genus [14]. Due to the absence of a *coxA* gene standard in bacterial classification, divergences of *coxA* sequences in *Rickettsia* were used to evaluate those between SMLS and *O. tsutsugamushi*. All 29 rickettsial *coxA* sequences were downloaded from GenBank on 25 Jan 2011 (Table S1). The uncorrected p-distance between the *coxA* sequences of SMLS and *O. tsutsugamushi* was 0.207, and this was much larger than the largest p-distance within *Rickettsia* (0.171 for *R. bellii* vs. *R. prowazekii*; Table S1). Assuming the divergence in *coxA* sequences of *Rickettsia* reflected intrageneric variation in the family Rickettsiaceae, then both *coxA* and *16S rRNA* divergences between SMLS and *O. tsutsugamushi* reached an intergeneric level.

*In situ* hybridization revealed that SMLS was housed in two types of embryonic cells—sheath cell and secondary bacteriocytes—both of which were located near primary bacteriocytes that contained *B. aphidicola*. This discovery implied a probable interaction between SMLS and *B. aphidicola*. Further, SMLS was also detected in hemolymph. The *in vivo* localizations were very similar to those of other, thoroughly investigated aphid secondary symbionts including *S. symbiotica*, *H. defensa*, *R. insecticola* and *Rickettsia* [7], [12], [20], [36]. Although speculative, the same mechanisms of infection, proliferation and vertical transmission may be shared by SMLS and the other secondary symbionts. *In vivo* localizations indicate that aphid secondary symbionts may have identical traits. Herein, ZK-strain aphids are discovered to be infected with SMLS only; no infection of *Rickettsia*, the other three main secondary symbionts of aphids and *W. pipientis* is detected. The two controls confirmed the hybridization's specificity. However, the probe target SMLS used in present study was designed referring to the one target *Rickettsia*, its use in distinguishing *Rickettsia* and SMLS must be taken with caution.

In general, the population of SMLS and *B. aphidicola* exhibit different developmental dynamics in their hosts. *Buchnera aphidicola* provides nutrition essential for aphid survival, particularly for the rapid production of embryos [1]. The population dynamics of *B. aphidicola* appear to be typical of aphids, as evidenced by patterns in pea aphids [20], [37]; the symbiont's density increases during nymphal growth, peaks during the active reproduction of young adults and declines in older stages. The resurgence of *B. aphidicola* at the 13 day-stage is probably due to the mismatch of rates of proliferation and consumption. When normalized with host gene titers, the density of *B. aphidicola* declines during the 5 to 9 day-stages. Apparently, *B. aphidicola*'s proliferation cannot match the rapid growth of young aphids in those stages. In comparison, the population of SMLS exhibits an increase-decline-increase curve, and the highest density occurs at the 29 day-stage, the last day-stage examined herein. Moreover, the same density dynamic is obtained after normalization with host gene titers. Two other aphid secondary symbionts (*S. symbiotica* and *Rickettsia*) have population dynamics that differ from that of *B. aphidicola* [20], [37]. Whereas, the infection level of *Rickettsia* maintains in older aphids, the population of *S. symbiotica* increases in older aphids and this is coincident with that of SMLS. Thus, whereas *B. aphidicola* is an obligate symbiont of aphids, the secondary symbiotic relationship of SMLS differs. This difference may drive the divergent population dynamics.

In addition to *S. miscanthi*, only the two wheat-feeding species (*S. graminum* and *R. padi*) among 13 tested species of aphids appear to be infected by SMLS, and no infection is obtained for *Rickettsia*. All three strains of SMLS have identical *16S rRNA* sequences suggesting a recent horizontal transmission among the three wheat-feeding aphids. Secondary symbionts can be transferred between species of aphids [11], [12], yet the mechanisms of these interspecific transmissions remains undiscovered [38]. *Wolbachia pipientis* may be transferred via feeding on plants [39], [40]. Because all of the three SMLS-infected aphids feed on wheat, it is possible that either feeding habits or wheat seedlings are responsible for SMLS transmission.

We collected fresh wheat seedlings and those that had been fed to aphids of the ZK-strain and then froze them in liquid nitrogen. Extracted bacterial total DNA was subjected to *16S rRNA* diagnostic PCR. SMLS was not detected on either fresh wheat seedlings or those that had been fed to ZK-strain aphids. Thus, wheat seedlings could not be associated with the transmission of SMLS. Another route must have been responsible for the horizontal transmission of SMLS among wheat-feeding aphids.

We could not test whether SMLS specifically infected wheat-feeding aphids only or not. A large-scale survey of SMLS in aphids was not possible, and infection rates of SMLS within host species vary with geography, as documented in *R. padi* (Table 1) and *S. miscanthi* [14]. These tests would have required wide-scale sampling, both taxonomically and geographically. Regardless of why, SMLS widely infected wheat-feeding aphids.

In insects, vertically transmitted bacteria promote their transmission either by manipulating their host's reproduction (e.g. *W. pipientis*) [41], or by increasing the fitness of infected hosts (e.g. *S. symbiotica*, *H. defensa*, and *R. insecticola*) [15], [16], [17], [18]. SMLS is vertically transmitted from mother to offspring with high fidelity, at least under laboratory rearing conditions. *Sitobion miscanthi* is largely parthenogenetic making it is unlikely that SMLS spreads by manipulating the reproductive systems of *S. miscanthi*, as *W. pipientis* does in arthropods. Further studies are required to investigate whether or not SMLS infections increase the fitness of *S. miscanthi*.

## Supporting Information

Table S1 Matrix of uncorrected p-distance of *coxA* sequences in genus *Rickettsia*.
(DOC)
