# Supplementary material for: Cellular Tropism, Population Dynamics, Host Range and Taxonomic Status of an Aphid Secondary Symbiont, SMLS (Sitobion miscanthi L Type Symbiont)
Source: PLoS One. 2011 Jul 15;6(7):e21944. doi: 10.1371/journal.pone.0021944 (PMC3137594; doi:10.1371/journal.pone.0021944)
Supplement: Table S1 — Matrix of uncorrected p-distance of coxA sequences in genus Rickettsia. (DOC) [file pone.0021944.s001.doc]

Table S1. Uncorrected p-distance of *coxA* sequences in genus *Rickettsia*.

|  | 1 | 2 | 3 | 4 | 5 | 6 | 7 | 8 | 9 | 10 | 11 | 12 | 13 | 14 | 15 | 16 | 17 | 18 | 19 | 20 | 21 | 22 | 23 | 24 | 25 | 26 | 27 | 28 | 29 |
| --- | --- | --- | --- | --- | --- | --- | --- | --- | --- | --- | --- | --- | --- | --- | --- | --- | --- | --- | --- | --- | --- | --- | --- | --- | --- | --- | --- | --- | --- |
| 1 |  |  |  |  |  |  |  |  |  |  |  |  |  |  |  |  |  |  |  |  |  |  |  |  |  |  |  |  |  |
| 2 | 0.139 |  |  |  |  |  |  |  |  |  |  |  |  |  |  |  |  |  |  |  |  |  |  |  |  |  |  |  |  |
| 3 | 0.138 | 0.171 |  |  |  |  |  |  |  |  |  |  |  |  |  |  |  |  |  |  |  |  |  |  |  |  |  |  |  |
| 4 | 0.013 | 0.141 | 0.137 |  |  |  |  |  |  |  |  |  |  |  |  |  |  |  |  |  |  |  |  |  |  |  |  |  |  |
| 5 | 0.076 | 0.141 | 0.120 | 0.076 |  |  |  |  |  |  |  |  |  |  |  |  |  |  |  |  |  |  |  |  |  |  |  |  |  |
| 6 | 0.099 | 0.135 | 0.141 | 0.100 | 0.100 |  |  |  |  |  |  |  |  |  |  |  |  |  |  |  |  |  |  |  |  |  |  |  |  |
| 7 | 0.088 | 0.137 | 0.138 | 0.007 | 0.073 | 0.098 |  |  |  |  |  |  |  |  |  |  |  |  |  |  |  |  |  |  |  |  |  |  |  |
| 8 | 0.016 | 0.132 | 0.138 | 0.007 | 0.075 | 0.104 | 0.084 |  |  |  |  |  |  |  |  |  |  |  |  |  |  |  |  |  |  |  |  |  |  |
| 9 | 0.009 | 0.136 | 0.135 | 0.015 | 0.071 | 0.098 | 0.013 | 0.081 |  |  |  |  |  |  |  |  |  |  |  |  |  |  |  |  |  |  |  |  |  |
| 10 | 0.123 | 0.139 | 0.139 | 0.009 | 0.073 | 0.098 | 0.006 | 0.083 | 0.013 |  |  |  |  |  |  |  |  |  |  |  |  |  |  |  |  |  |  |  |  |
| 11 | 0.121 | 0.072 | 0.142 | 0.127 | 0.124 | 0.110 | 0.123 | 0.132 | 0.116 | 0.120 |  |  |  |  |  |  |  |  |  |  |  |  |  |  |  |  |  |  |  |
| 12 | 0.121 | 0.071 | 0.142 | 0.125 | 0.123 | 0.112 | 0.121 | 0.134 | 0.114 | 0.119 | 0.001 |  |  |  |  |  |  |  |  |  |  |  |  |  |  |  |  |  |  |
| 13 | 0.121 | 0.071 | 0.142 | 0.125 | 0.123 | 0.112 | 0.121 | 0.134 | 0.114 | 0.119 | 0.001 | 0.000 |  |  |  |  |  |  |  |  |  |  |  |  |  |  |  |  |  |
| 14 | 0.121 | 0.025 | 0.151 | 0.124 | 0.123 | 0.119 | 0.120 | 0.128 | 0.113 | 0.120 | 0.078 | 0.076 | 0.076 |  |  |  |  |  |  |  |  |  |  |  |  |  |  |  |  |
| 15 | 0.121 | 0.025 | 0.151 | 0.124 | 0.123 | 0.119 | 0.120 | 0.128 | 0.113 | 0.120 | 0.078 | 0.076 | 0.076 | 0.000 |  |  |  |  |  |  |  |  |  |  |  |  |  |  |  |
| 16 | 0.076 | 0.119 | 0.116 | 0.076 | 0.078 | 0.104 | 0.074 | 0.033 | 0.067 | 0.074 | 0.129 | 0.128 | 0.128 | 0.124 | 0.124 |  |  |  |  |  |  |  |  |  |  |  |  |  |  |
| 17 | 0.078 | 0.119 | 0.121 | 0.078 | 0.079 | 0.104 | 0.075 | 0.029 | 0.068 | 0.075 | 0.129 | 0.128 | 0.128 | 0.121 | 0.121 | 0.023 |  |  |  |  |  |  |  |  |  |  |  |  |  |
| 18 | 0.079 | 0.127 | 0.117 | 0.080 | 0.090 | 0.093 | 0.078 | 0.046 | 0.074 | 0.075 | 0.132 | 0.131 | 0.131 | 0.129 | 0.129 | 0.050 | 0.040 |  |  |  |  |  |  |  |  |  |  |  |  |
| 19 | 0.079 | 0.119 | 0.120 | 0.079 | 0.078 | 0.102 | 0.076 | 0.030 | 0.069 | 0.076 | 0.128 | 0.127 | 0.127 | 0.121 | 0.121 | 0.025 | 0.001 | 0.041 |  |  |  |  |  |  |  |  |  |  |  |
| 20 | 0.091 | 0.113 | 0.139 | 0.094 | 0.105 | 0.110 | 0.091 | 0.112 | 0.087 | 0.087 | 0.124 | 0.125 | 0.125 | 0.124 | 0.124 | 0.101 | 0.105 | 0.112 | 0.105 |  |  |  |  |  |  |  |  |  |  |
| 21 | 0.091 | 0.113 | 0.135 | 0.095 | 0.106 | 0.113 | 0.091 | 0.113 | 0.086 | 0.089 | 0.124 | 0.125 | 0.125 | 0.124 | 0.124 | 0.102 | 0.106 | 0.110 | 0.106 | 0.007 |  |  |  |  |  |  |  |  |  |
| 22 | 0.093 | 0.113 | 0.140 | 0.095 | 0.104 | 0.110 | 0.093 | 0.110 | 0.086 | 0.089 | 0.124 | 0.125 | 0.125 | 0.124 | 0.124 | 0.099 | 0.104 | 0.110 | 0.104 | 0.004 | 0.005 |  |  |  |  |  |  |  |  |
| 23 | 0.093 | 0.113 | 0.140 | 0.095 | 0.104 | 0.110 | 0.093 | 0.110 | 0.086 | 0.089 | 0.124 | 0.125 | 0.125 | 0.124 | 0.124 | 0.099 | 0.104 | 0.110 | 0.104 | 0.001 | 0.005 | 0.003 |  |  |  |  |  |  |  |
| 24 | 0.093 | 0.113 | 0.140 | 0.095 | 0.104 | 0.110 | 0.093 | 0.110 | 0.086 | 0.089 | 0.124 | 0.125 | 0.125 | 0.124 | 0.124 | 0.099 | 0.104 | 0.110 | 0.104 | 0.004 | 0.005 | 0.000 | 0.003 |  |  |  |  |  |  |
| 25 | 0.094 | 0.112 | 0.140 | 0.097 | 0.104 | 0.108 | 0.094 | 0.110 | 0.087 | 0.090 | 0.123 | 0.124 | 0.124 | 0.123 | 0.123 | 0.101 | 0.105 | 0.112 | 0.105 | 0.005 | 0.007 | 0.004 | 0.004 | 0.004 |  |  |  |  |  |
| 26 | 0.097 | 0.117 | 0.144 | 0.099 | 0.108 | 0.114 | 0.097 | 0.114 | 0.090 | 0.093 | 0.128 | 0.129 | 0.129 | 0.128 | 0.128 | 0.104 | 0.108 | 0.114 | 0.108 | 0.008 | 0.010 | 0.007 | 0.007 | 0.007 | 0.008 |  |  |  |  |
| 27 | 0.097 | 0.113 | 0.140 | 0.099 | 0.105 | 0.113 | 0.097 | 0.112 | 0.090 | 0.093 | 0.124 | 0.125 | 0.125 | 0.124 | 0.124 | 0.102 | 0.106 | 0.113 | 0.106 | 0.008 | 0.010 | 0.007 | 0.007 | 0.007 | 0.005 | 0.005 |  |  |  |
| 28 | 0.120 | 0.132 | 0.157 | 0.124 | 0.131 | 0.125 | 0.121 | 0.135 | 0.120 | 0.120 | 0.136 | 0.135 | 0.135 | 0.135 | 0.135 | 0.134 | 0.136 | 0.134 | 0.138 | 0.134 | 0.134 | 0.134 | 0.134 | 0.134 | 0.129 | 0.138 | 0.135 |  |  |
| 29 | 0.120 | 0.023 | 0.150 | 0.123 | 0.121 | 0.117 | 0.119 | 0.127 | 0.112 | 0.119 | 0.076 | 0.075 | 0.075 | 0.001 | 0.001 | 0.123 | 0.120 | 0.128 | 0.120 | 0.123 | 0.123 | 0.123 | 0.123 | 0.123 | 0.121 | 0.127 | 0.123 | 0.134 |  |

1, *Rickettsia rickettsii*-NC_010263; 2, *Rickettsia bellii*-NC_007940; 3, *Rickettsia prowazekii*-NC_000963; 4, *Rickettsia africae*-CP001612; 5, *Rickettsia akari*-CP000847; 6, *Rickettsia canadensis*-CP000409; 7, *Rickettsia conorii*-AE006914; 8, *Rickettsia felis*-CP000053; 9, *Rickettsia massiliae*-CP000683;

10, *Rickettsia peacockii*-CP001227; 11, Rickettsia of Bombyliid bee fly species strain WOL150653-FJ666778; 12, *Rickettsia* of Bombyliid bee fly species strain WOL150801-FJ666776; 13, *Rickettsia* of Acyrthosiphon pisum strain PAR-FJ666777; 14, *Rickettsia* of Elaterid beetle species strain PNG0392- FJ666781;

15, *Rickettsia* of Noctuid moth species strain PNG0405-FJ666782; 16, *Rickettsia* of Cercopidae species spittlebug strain Y-FJ666791; 17, *Rickettsia* of *Aulogymnus balani*/*skianeuros* strain ABSWASP-FJ666793; 18, *Rickettsia* of *Pediobius rotundatus* strain PRWASP-FJ666794; 19, *Rickettsia* of *Aulogymnus trilineatus* strain ATWASP107- FJ666792; 20, *Rickettsia* of *Adalia bipunctata* strain 9I-FJ666786; 21, *Rickettsia* of *Subcoccinella vigintiquatuorpunctata* strain J-FJ666783; 22, *Rickettsia* of *Adalia decempunctata* strain Y-FJ666790; 23, *Rickettsia* of *Adalia bipunctata* strain 10J-FJ666787; 24, *Rickettsia* of *Adalia bipunctata* strain 10J- FJ666785; 25, *Rickettsia* of *Scymnus frontalis* strain THETA-FJ666784; 26, *Rickettsia* of *Calvia quatuordecimguttata* strain I-FJ666789; 27, *Rickettsia* of Halyzia 16-guttata orange ladybird strain D-FJ666788; 28, *Rickettsia* of Meloidae species strain 212489-FJ666775; 29, *Rickettsia* of *Coccidula rufa* strain H- FJ666795. Uncorrected p-distance was calculated in MEGA 4.0, the gaps and missing data in sequences alignment were pairwise deleted. The largest divergence occurred between *Rickettsia bellii* and *Rickettsia prowazekii*, which highlighted in RED.
